# Time to halt perioperative chemotherapy for resectable colorectal liver metastasis?

**DOI:** 10.1093/bjs/znab425

**Published:** 2021-12-07

**Authors:** Kjetil Søreide

**Affiliations:** Department of Gastrointestinal Surgery, Hepatopancreatobiliary Unit, Stavanger University Hospital and University of Bergen, Norway

Colorectal liver metastasis (CRLM) is a treatable condition, with the prospect of cure in some patients in whom surgery and multimodal therapy is possible^1–3^. Historical boundaries to liver resection (such as number, size, and location of CRLMs) have been replaced by poor cancer biology as the principal prognostic factor^4–7^. Before liver resection, surgeons now assess what will stay (future liver remnant), rather than contemplating what will go (tumour burden), to determine surgical operability or need for preoperative modifications (for example, portal vein embolization or associating liver partition and portal vein ligation for staged hepatectomy) to increase resectability and prevent liver failure^3^. Further technical advances are being introduced, such as simultaneous portal vein and liver vein embolization (liver vein deprivation)^8^, and are now being assessed in ongoing prospective randomized trials. Systemic cytotoxic therapy (such as oxaliplatin or irinotecan), biological agents (bevacizumab or cetuximab), and immune checkpoint inhibitors have expanded systemic treatment options such that tumours are deemed suitable for resection^9^. Unfortunately, the vast majority (70–80 per cent) experience recurrence after hepatectomy, most often in the liver (regrowth of persistent CRLMs)^10^. Repeat liver resection with or without ablation techniques^2^, hepatic artery infusion^11^, and selective internal radiation therapy^12^ or drug delivery are all possible for disease control. There are reports of improved progression-free survival but there may be no gain in overall survival, so the selection of patients is key to limiting what could be non-effective or even futile interventions.

One of the most controversial aspects of CRLM management is perioperative chemotherapy. Of note, the assumed effect of chemotherapy is extrapolated and based on studies of stage III adjuvant and unresectable CRLMs. Novel chemotherapy regimens were tested in patients with resectable CRLMs^13^, but no effect on overall survival was found in the EPOC trial. The resection criteria for CRLMs at the time are now out of date (maximum of 4 metastases). Further confusion was added by the New EPOC trial^14^, in which cetuximab (an epidermal growth factor receptor inhibitor that is effective in the absence of *RAS* pathway mutations) added to perioperative treatment for operable CRLM was associated with worse overall survival^15^. The New EPOC study also included mostly low-burden disease (about 80 per cent had 3 or fewer lesions). The cumulative evidence does not support postoperative chemotherapy after CRLM resection^16^, yet it is included in guidelines (European and North American) and is considered standard of care in some centres around the world^17^.

More recently, the JCOG0603 trial^18^ found that overall survival was no better with oxaliplatin-based therapy after CRLM surgery. Although this trial had no limit on the number of CRLMs (if resectable), the vast majority of patients enrolled had oligometastatic disease (3 or fewer lesions), yet needed dose reduction or had some form of dose delay. Indeed, only two-thirds completed the intended nine cycles of chemotherapy. The 3-year survival rate of 91.8 per cent suggests that metastasectomy alone provides excellent disease control in selected patients.

If there is no overall survival gain from adjuvant chemotherapy in this setting, why is overtreatment (no proven or only modest benefits exist, yet side-effect risks remain) so pervasively persistent? As in much of healthcare, the clinician’s desire to do something is much stronger than the decision to do nothing, fed by impressions that more is better. The JCOG0603 trial took 13 years, with slow accrual likely reflecting patients and clinicians being hesitant to randomize to hepatectomy alone as though it was intuitively inferior. Meanwhile half of patients receiving oxaliplatin have evidence of neurotoxicity at 1 year, with symptoms persisting in one-quarter of patients^19^. In the JCOG0603 trial^18^, most had some neuropathic symptoms and 10 per cent had severe neuropathy. If adjuvant chemotherapy over surgery alone for liver-limited disease (4 or fewer CRLMs) cannot improve survival, we should pause and reflect. Have we asked patients what they want? How is it best to design trials of tailored treatment when current risk scores predict outcomes so inaccurately?^6^^,^^7^ Why is primary tumour sideness^20^ such a strong predictor of overall survival? Better therapy may emerge as we learn from the molecular mimicry, heterogeneity, and clonal evolution of tumours that occur in the treatment journey. As it stands, the liberal use of perioperative chemotherapy in patients with resectable CRLM should be questioned.

*Disclosure*. The author declares no conflict of interest.
